# Drone Detection and Classification Using Physical-Layer Protocol Statistical Fingerprint

**DOI:** 10.3390/s22176701

**Published:** 2022-09-05

**Authors:** Louis Morge-Rollet, Denis Le Jeune, Frédéric Le Roy, Charles Canaff, Roland Gautier

**Affiliations:** 1 ENSTA Bretagne, Lab-STICC, CNRS, UMR 6285, F-29200 Brest, France; 2 Université de Bretagne Occidentale (UBO, Brest Campus), Lab-STICC, CNRS, UMR 6285, F-29200 Brest, France

**Keywords:** drone detection, drone classification, RF sensing, physical-layer authentication

## Abstract

We propose a novel approach for drone detection and classification based on RF communication link analysis. Our approach analyses large signal record including several packets and can be decomposed of two successive steps: signal detection and drone classification. On one hand, the signal detection step is based on Power Spectral Entropy (PSE), a measure of the energy distribution uniformity in the frequency domain. It consists of detecting a structured signal such as a communication signal with a lower PSE than a noise one. On the other hand, the classification step is based on a so-called physical-layer protocol statistical fingerprint (PLSPF). This method extracts the packets at the physical layer using hysteresis thresholding, then computes statistical features for classification based on extracted packets. It consists of performing traffic analysis of communication link between the drone and its controller. Conversely to classic drone traffic analysis working at data link layer (or at upper layers), it performs traffic analysis directly from the corresponding I/Q signal, i.e., at the physical layer. The approach shows interesting properties such as scale invariance, frequency invariance, and noise robustness. Furthermore, the classification method allows us to distinguish WiFi drones from other WiFi devices due to underlying requirement of drone communications such as good reactivity in control. Finally, we propose different experiments to highlight theses properties and performances. The physical-layer protocol statistical fingerprint exploiting communication specificities could also be used in addition of RF fingerprinting method to perform authentication of devices at the physical-layer.

## 1. Introduction

Nowadays, drones have found tremendous usages such as food delivery, building inspections and hobbyist interests. However, unregulated use of amateur-UAV cause important security concerns. Over the past few years several incidents happened implying micro-UAVs such as commercial drones. Besides privacy concern due to drones, it also causes other security problems of intrusion in sensitive facilities such as airports and nuclear power plants. Particulary, in 2017 during a presentation at MobySys’17 [1], Pr. Nguyen shows how a collision with a drone can be destructive for an airplane during flight. Other examples are widely discussed in the literature [1,2,3].

Drone detection and classification is increasingly becoming an important field of scientific publications. Several techniques exists for drone detection and classification using different media such as video, sound, radar and RF [2,3]. Furthermore, drone neutralization is also an important topic. Several techniques can be used for drone neutralization [4], some are non-destructive such as those using RF jammer or GPS spoofer and others are destructive such as high power microwave devices or cinetic weapons. However, drone neutralization techniques will not be addressed in this paper.

This article proposes several innovative ideas: signal detection using Power Spectral Entropy (PSE), drone classification using physical-layer protocol statistical fingerprint (PLPSF). We also use data augmentation by noise injection method allowing us to evaluate the mentioned methods with variable Signal-to-Noise Ratio (SNR). Finally, we propose a statistical analysis method to evaluate robustness of our packets extraction method to the environmental conditions. The Section 2 expose the state of the art in drone detection/classification including video, sound, radar, RF and WiFi-based. The Section 3 presents the methodology including noise injection, detection and classification. The Section 4 reports our different experiments with different UAVs allowing to highlight the performances of the presented methods. The Section 5 is divided in two part: the first part is a discussion about using PLPSF as a physical-layer authentication method and the second part presents the perspectives of our work. We finally conclude with Section 6 which synthesizes the work done.

## 2. State of the Art

Drone detection and classification is a difficult problem due to furtive aspect of drones such as small dimensions. This section compares different detection and classification methods and presents the pros/cons.

### 2.1. Video-Based

Video-based methods are passive and depend on optical sensors, particularly camera, to detect and classify the drone by visual aspects. However, these techniques are limited by distance range, luminosity conditions and line of sight. Furthermore, there is strong problems of false alarm due to birds presence. Temperature can also be used to detect using optical sensors sensitive to infrared signatures [5]. However, these techniques are generally different from classic video-based techniques and are dealt separately in the literature because they classically concern turbo-jet drones.

### 2.2. Sound-Based

Sound-based methods try to detect and classify drones using acoustic signatures. As video-based techniques, sound-based detection/classification techniques are also passive methods involving a microphone or microphone array. Indeed, drones have specific acoustic signatures due to propellers creating high-pitch sounds [5]. Sound-based techniques are sensitive to environmental noises and the distance range.

### 2.3. Radar-Based

Radar-based methods includes detecting and classifying drones using emitted electromagnetic wave. Contrary to previously introduced methods, radar-based methods are active, i.e., they require emission of electromagnetic wave to work. Generally, radar used the backscattering of the emitted wave to detect the target (position, speed, …). Moreover, drone detection/classification using radar-based techniques can also exploit micro-Doppler effect [6,7], including effect on electromagnetic waves due to propellers vibrations. Radar-based techniques are sensitive to small radar cross surface (RCS) and can be perturbated by birds presence just as video-based techniques.

### 2.4. RF-Based

Radio frequency methods consist of detecting and classifying drones using different communication links: controller link, video link and telemetry link [8,9,10]. Furthermore, it can be used in conjunction with goniometry method to estimate drone position [11]. These techniques are passive and require at least the presence of one of the drone links. Despite this major drawback, RF sensing does not suffer from line-of-sight problematic and works with relatively long distance range. Active RF-sensing techniques also exist in the literature such as [12,13,14].

### 2.5. WiFi-Based

Wifi-based methods are subcases of radio frequency based techniques focusing on detecting and classifying WiFi drones [5,15,16]. WiFi drones are shown to have a different statistical signatures at the data link layer (second layer of OSI model) than other WiFi devices. Indeed, WiFi drones need to communicate often with controllers to ensure accurate control [12]. In addition to previously introduced disadvantages, WiFi-based methods are also protocol specific.

### 2.6. Fusion-Based

Several industrial products for drone detection/classification use a fusion of previously introduced methods such as Thales EagleSHIELD [17]. Despite increasing products cost due to more complex integration, it allows to sum up the different advantages and compensate different disadvantages. These systems can also include drones neutralization technologies.

For interested readers, several articles provide much complete review of drone detection and classification techniques [2,3].

## 3. Methodology

The methods presented in this paper are RF-based approaches; they allow us to detect and classify drones exploiting the baseband signals of RF links using a low-cost RF recorder. Furthermore, the proposed classification method is inspired from WiFi-based method exploiting protocol statistical fingerprint [5,15]. However, compared to those methods exploiting statistical fingerprint at data link layer which are protocol specific (WiFi), our method exploits the same protocol statistical fingerprint but at physical layer, thus becoming protocol agnostic.

### 3.1. Global Architecture

**Figure 1:**
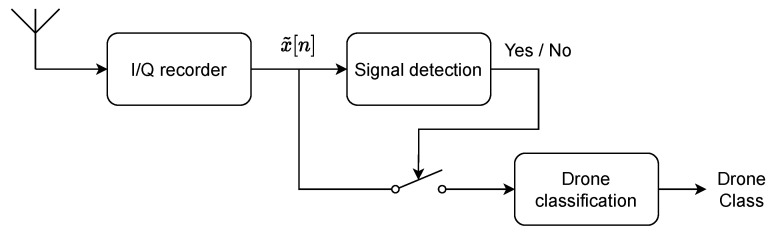
Global architecture.

The global architecture of proposed signal detection and drone classification method is presented in Figure 1. One advantage of our methodology is to analyses a signal window without knowledge about signal presence. The window extraction is based on a low-cost signal recorder extracting baseband signal from a specific frequency band during a certain amount of time, called signal window. After recording, the signal window is analysed to determine the presence of structured signals and classification is applied if a signal is detected. Thus, this methodology can be used to scan several bands for drone detection and classification.

### 3.2. Dataset

The dataset used for this study is composed of several drones but also includes WiFi records coming from classic communications (smartphone, …). The baseband signals are recorded using SignalHound BB60C (low-cost I/Q signal recorder) with VERT2450 antenna at 20 Msamples/s in 2.4 and 5 GHz ISM bands. The BB60C has a 27 MHz instantaneous bandwidth due to an analog filter. The baseband signal is sampled at 40 MSamples/s, then filtered by 20 MHz numerical low-pass filter and decimate by a factor 2. To do so, the central frequency is manually centered on communication signal (preferably on the video link) using spectrogram and the corresponding I/Q signal is recorded. Then, the radio recordings are divided in 100 ms non-overlapping segments of baseband signal ready for processing. The segment size was chosen accordingly to include several drone packets but also sufficient to capture at least one WiFi beacon. Indeed, WiFi beacons between access point and mobile devices are usually exchanged every 100 ms [12]. Furthermore, the segment size does not exceed 100 ms to avoid problems when extracting packets under a variable Received Signal Strength Indication (RSSI). The drones which compose the dataset are described in Table 1. For each drone class described in this table, we performed two independent 10 seconds recordings. WiFi records are composed of two independent 5 s recordings. The first recordings of each class are reserved for training (called training recording) while the second recordings of each class are reserved for testing (called testing recording). This methodology allows us to evaluate drone detection/classification close to real implementation conditions. A signal record contain the communication link between the drone and its controller (in the same band), i.e., this link could be composed of the controller link, the video link and even the telemetry link.

**Figure 2:**
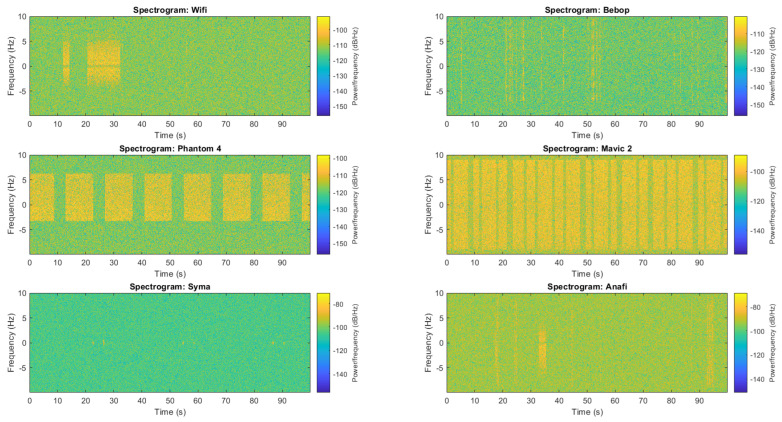
Spectrogram of drones signals.

Figure 2 show the spectrograms of the different drones composing the dataset with a SNR of 3 dB. We can observe that some drones have really repetitive temporal behaviours such as Phantom 4 Pro, Mavic 2 Pro and Syma X5C. Conversely, the WiFi drones (Parrot Bebop, Parrot Anafi) have less structured temporal behaviours that other drones but emit more frequently than other WiFi devices [12]. Thus, this figure shows the advantage of extracting the temporal behaviour over a signal window to perform drone classification.

### 3.3. Noise Injection

This step is necessary to perform data augmentation as presented by Soltani et al. in [18], allowing us to test the noise robustness of our algorithms. This pre-processing allows to inject noise at a specific power to obtain a desired signal-to-noise ratio ($SNR_{d}$) compared to experimental approaches where SNR is less controlled. The noise injection pre-processing step requires to estimate the noise power $P_{n}$ and the signal+noise power $P_{sn}$ as described in [19]. For this step, we use a low-pass filter with 10 kHz bandwidth to filter the signal envelope (absolute value of the signal) then we extract packets using classic thresholding to determine the both parts. The threshold correspond to the mean between the lowest value of the signal envelope filtered and of the highest value of the signal envelope filtered. Once the different parts are extracted, $P_{n}$ and $P_{sn}$ are computed and the signal power $P_{s}$ is estimated by $P_{s}=P_{sn}-P_{n}$. Using $P_{s}$ and $P_{n}$, the SNR is then computed and the power of noise to inject is obtained as following $P_{n^{\prime }}=\frac{P_{s}}{SNR_{d}}-P_{n}$. Finally, the noise to inject is generated as a additive gaussian complex white noise with power equal to $P_{n^{\prime }}$ and it is added to the signal to obtain the desired SNR.

### 3.4. Signal Detection: Power Spectral Entropy (PSE)

Signal detection is the second step before drone classification. Classical signal detection techniques are the following [8]: energy detection, matched filter, cyclostationarity and eigenvalue methods. More specific methods have been proposed for drone detection such as Markov chain detector [20] based on energy transition. In our case, we want to detect signal presence in relatively large signal window with low computation complexity, independently of temporal signal location and without knowledge about signal of interest. Thus, our detection method is based on power spectral entropy (PSE) of baseband signal $\tilde{x}\left[n\right]$, a measure of energy distribution uniformity in frequency domain. It consists in considering power spectral density (PSD) as a probability density and to compute the entropy on this empirical distribution. Theoretically, the PSE is maximized by white noise because it has uniform frequency distribution and then maximizes entropy. Therefore, PSE can be used to differentiate white noise from more structured signals such as drone communications signals. However, this detection approach could also be applied to more realistic noise such as background noise.

For this purpose, we directly process the baseband signal $\tilde{x}\left[i\right]$ with i∈〚0;L−1〛 (with *L* the signal length). The different steps for detecting signal using spectral entropy are:

1. The first step estimate PSD P(i) with i∈〚0;N−1〛, we choose Bartlett estimator [21] (N = 2048, $K=\left\lfloor \frac{L}{N}\right\rfloor$) instead of periodogram due to its consistency properties.
  $$P\left(i\right)=\frac{1}{K}\sum_{k=0}^{K-1}(\frac{1}{N}|\sum_{n=0}^{N-1}\tilde{x}\left(n+kN\right)e^{-2j\pi \frac{ni}{N}}{|}^{2})$$
2. Then normalize the PSD to obtain the so-called frequency probability density function (FPDF) $p_{i}$.
  $$p_{i}=\frac{P(i)}{\sum_{i}P\left(i\right)}$$
3. After estimating the FPDF, we compute the entropy to obtain the PSE.
  $$\mathrm{PSE}=\sum_{i=0}^{N-1}p_{i}log_{2}\left(1/p_{i}\right)$$
4. Finally, we compare the PSE to a specific threshold η (computed for a specific false alarm rate) to determine if it correspond to a noise or a signal.
  PSE⋛η

The computation complexity of signal detection step is O(LlogN). Indeed, a periodogram can be obtained using a fast fourier transform with a computation complexity of O(NlogN). Furthermore, the Bartlett estimator is composed of *K* periodograms and the resulting complexity is O(KNlogN): O(LlogN).

### 3.5. Drone Classification: Physical-Layer Protocol Statistical Fingerprint (PLPSF)

**Figure 3:**
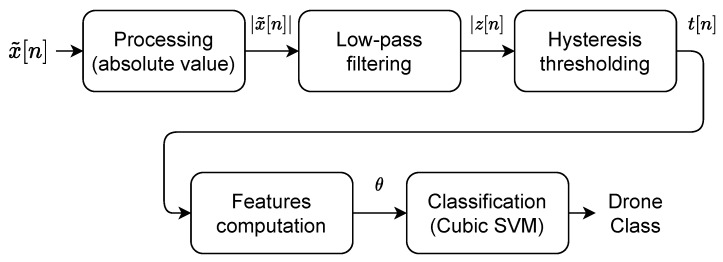
Structure of classification method.

This classification method is based on WiFi detection/classification algorithms using statistical fingerprint of communication packets/inter-packets duration. In [5,15], the authors compute statistical features at data link layer. For our part, we extract packets at the physical layer avoiding to be protocol specific by working directly on baseband signal $\tilde{x}\left[n\right]$. Our methodology is described in Figure 3. The first step compute the signal envelope $|\tilde{x}\left[n\right]|$. Then, we perform filtering using a low-pass filter h[i] ($F_{pass}=10$ kHz) allowing to only extract the low-frequency signal behaviour z[n] corresponding to the protocol information. We then perform hysteresis thresholding to extract packets as described by Algorithm 1. The hysteresis thresholding in inspired by works in computer vision such as Canny filtering [22] and require two thresholds: low threshold $\mu_{low}=0.5-\epsilon$, high threshold $\mu_{high}=0.5+\epsilon$ where ϵ is an hyperparameter. This thresholding technique avoid that a single packet is considered as several packets due to the energy drops during packets transmission. Statistical features are then computed on thresholded signal t[n] and classification is performed using Cubic Support Vector Machine classifier [23].

The statistical features θ are the following:

- Mean of packets duration (${\bar{m}}_{pck}$)
- Standard deviation of packets duration ($\sigma_{pck}$)
- Mean of inter-packets duration (${\bar{m}}_{ipck}$)
- Standard of inter-packets duration ($\sigma_{ipck}$)
- Number of packets ($N_{pck}$)

**Algorithm 1:** Hysteresis thresholding
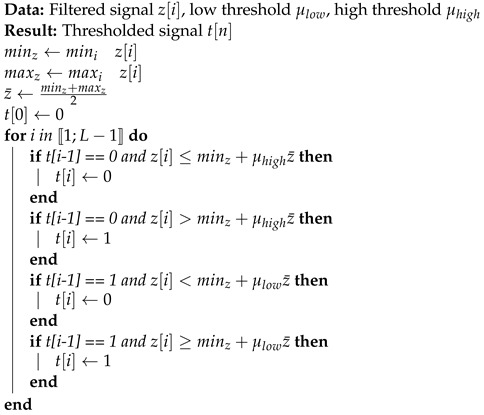


### 3.6. Invariances to Environmental Conditions

This subsection presents the invariances of the signal detection and drone classification methods that we presented. Invariances are important for the algorithms, because they prove the built-in robustness against different conditions (scale, rotation …) that detection and classification should not depend. For example, an image recognition algorithm must classify a cat picture regardless to its scale or its orientation. Particularly, the presented classification algorithm can be considered as a software-based approach contrary to RF Fingerprint approach [24]. Software-based approach corresponds to higher granularity level than RF Fingerprinting techniques. Indeed, as explained in [25], the software-based tries to differentiate devices from different make while ensuring devices from same make are classified in a same class. Therefore, the proposed classification method must be invariant to environmental conditions (Doppler shift, …) and impairments of same make devices (frequency offset, …) that does not contain information for drone classification. Concerning classification algorithm on signal s(t) there are several invariances (see Appendix A):

- Scale invariance $\tilde{y}\left(t\right)=a\tilde{x}\left(t\right)$: The algorithm is not sensitive to the complex coefficient a∈C and so makes the result invariant to homothety and phase rotation due to propagation and amplification. This can be performed thanks to the absolute value function allowing to remove any phase effect including phase rotation. Furthermore, covariant properties of filtering, minimum $min_{z}$, maximum $max_{z}$ and mean $\bar{z}$ computation allow homothety invariance.
- Frequency invariance $\tilde{y}\left(t\right)=\tilde{x}\left(t\right)e^{2j\pi \Delta ft}$: The algorithm is not sensitive to the frequency offset Δf due to frequency difference in oscillators (even in same make devices) and/or Doppler shifting. This is handled by absolute value allowing to remove any phase effect including frequency offset.

Furthermore, classification method have some robustness against impulsive noises. Indeed, low pass filter and hysteresis filtering avoid false alarms due to impulsive noises.

Detection algorithm has the same intrinsic invariances (see Appendix B): scale and frequency offset. Indeed, PSD normalization step allows scale invariance and entropy computation give invariance to frequency offset because entropy is not sensitive to central tendency of statistical distribution.

## 4. Experimentations

This section presents different results for the previously introduced detection and classification methods. All these results depend on the dataset presented in Section 3.2 and also rely on noise injection technique presented in Section 3.3.

### 4.1. Detection

**Figure 4:**
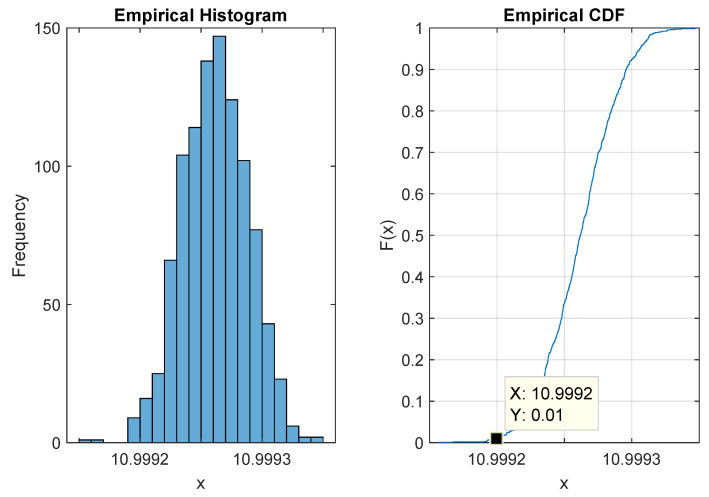
Histogram for PSE.

**Figure 5:**
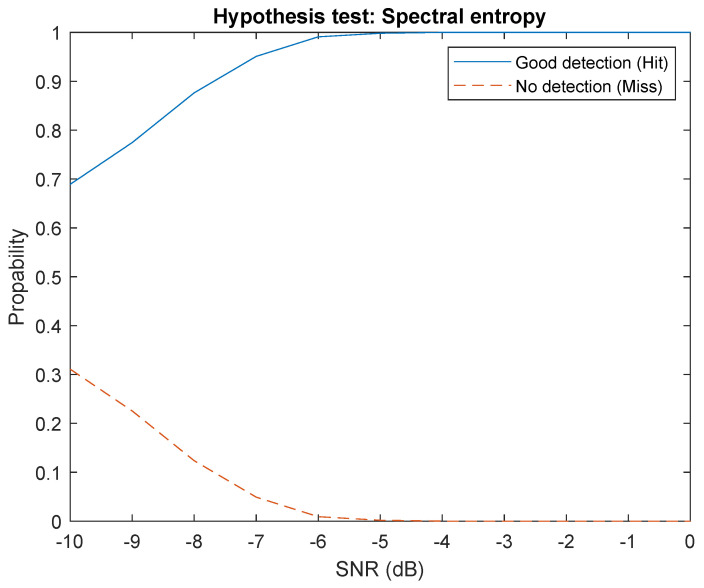
Results for detection.

The first step for the detection method is to compute the threshold η presented in Section 3.4. This consists of generating noise segments and computing the corresponding power spectral entropy. Then we sort the obtained spectral entropy measures of noise and select the threshold using a specific percentile (here 1%). As white noise maximize spectral entropy, we perform left unilateral hypothesis test, i.e., the reject region is [0;μ]. Once threshold μ is computed and the reject region is fixed, we use dataset signals, inject noise for a specific SNR, compute power spectral entropy, then perform hypothesis testing. The histogram of PSE and the correspond Cumulative Distribution Function (CDF) for hypothesis $H_{0}$ is show in Figure 4 and the threshold value μ is 10.9992. The results of the detection method in Figure 5 show good robustness against noise.

### 4.2. Statistical Robustness of Packets Extraction Method

To show good robustness to environmental conditions of our classification method, particularly of our packets extraction method, we perform statistical test under different conditions. The goal is to compare a reference sample (packet or inter-packet duration) of a specific class with a sample of the same class but dependent on specific environmental condition and conclude about the influence of the condition on the second sample. For that, we compute the empirical Cumulative Distribution Function F(x) of packet duration and inter-packet duration on the first record for each class. To achieve this, we split the signal record of specific class in 100 ms non-overlapping segments and inject noise to obtain a specific SNR (here 5 dB). Then, for each segments, we extract the different packet and inter-packet duration and aggregate it to obtain the packet duration and inter-packet duration samples of the whole signal. Finally, we compute the empirical Cumulative Distribution Function F(x) on packet duration sample and inter-packet duration sample. At the same time, for each class and for each different conditions, we split the first signal record in 100 ms non-overlapping segments, apply environmental conditions and inject noise to obtain specific SNR (dependent of environmental conditions). We reproduce the same steps (for each class) then previously to obtain the empirical Cumulative Distribution Function G(x) of packets duration and inter-packets duration for each environmental conditions. To show the impact of environmental conditions we perform a Kolmogorov–Smirnov test using this statistic $sup_{x}\left|F\left(x\right)-G\left(x\right)\right|$ (α = 5%). The different conditions for the statistical test are the following: (1) amplitude (5 dB), (2) different SNR (0 dB), (3) temporal shift (τ = 50 ms, 5 dB), (4) frequency offset (20 ppm, 5 dB).

We can observe in Table 2 and Table 3 the statistical robustness of our analysis for previously introduced invariances (amplitude and frequency offset) but also for temporal shifting. Although all *p*-values are not superior to 0.05, we can observe a certain robustness for lower SNR (0 dB) value of different records. Particularly for drones that does not present really structured and predictive packets and inter-packets duration, i.e., WiFi drones ((a) and (f)). Furthermore, a rejection of null hypothesis does not imply bad classification performance. This subsection shows statistical robustness to different condition for same class (intra-class variability) but classification differentiates devices from different makes (inter-class variability).

### 4.3. Classification

**Figure 6:**
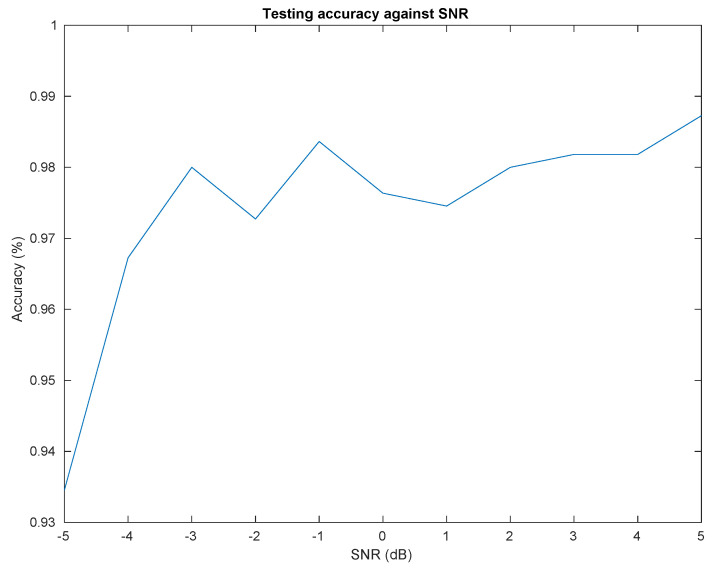
Classification rate against SNR.

**Figure 7:**
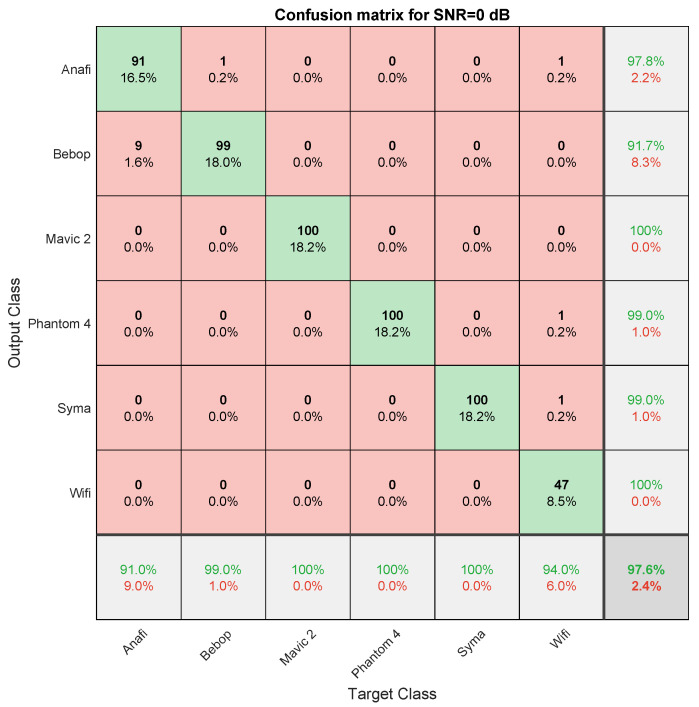
Confusion matrix (SNR = 0 dB). A green case corresponds to good classifications and a red case corresponds correspond to wrong classifications.

This section presents different performances of the drone classification method presented in Figure 3. Particularly, Figure 6 shows evolution of the classifier accuracy against different SNR values and Figure 7 presents a confusion matrix for a specific SNR value (SNR = 0 dB). The hyperparameters for hysteresis thresholding are the following: $\mu_{low}$ = 0.44 and $\mu_{high}$ = 0.56. As already explained in Section 3.2, the classifier is trained on training records and evaluate on testing records which are independently recorded signals. Furthermore, the SNR of training records is fixed to 5 dB while evaluation is performed depending of variable SNR. We can observe the performances stability to noise injection (SNR) which is a good property for long-range drone classification. We can also observe on Figure 7 that the majority of errors are made from ANAFI class to Bebop one, which makes sense because Bebop and ANAFI are both Parrot WiFi drones. Furthermore, some misclassifications are made from WiFi class due to the high variability of communications (access point, smartphone).

### 4.4. Parametric Analysis

For the statistical analysis and the drone classification performances evaluation, we considered that hyperparameters were fixed. In this subsection, we are interested in studying the influence of the hyperparameters on the accuracy but also to discuss about the potential advantages/disadvantages that change could produce. The packet extraction method depend of the following hyperparameters:

- Window size: The window size correspond to the segment size and is equal to 100 ms.
- Processing: The first step of packet extraction extract packet using signal envelope (f(s(t))=|s(t)|).
- Threshold: The hysteresis thresholding depend of two thresholds: $\mu_{low}=0.44$ and $\mu_{high}=0.56$ (ϵ=0.06).

The hyperparameters values chosen to evaluate the classification method allow relatively good accuracy as shown in the previous section but also present a certain robustness to variable RSSI as explained in the Section 3.2.

#### 4.4.1. Window Size

**Figure 8:**
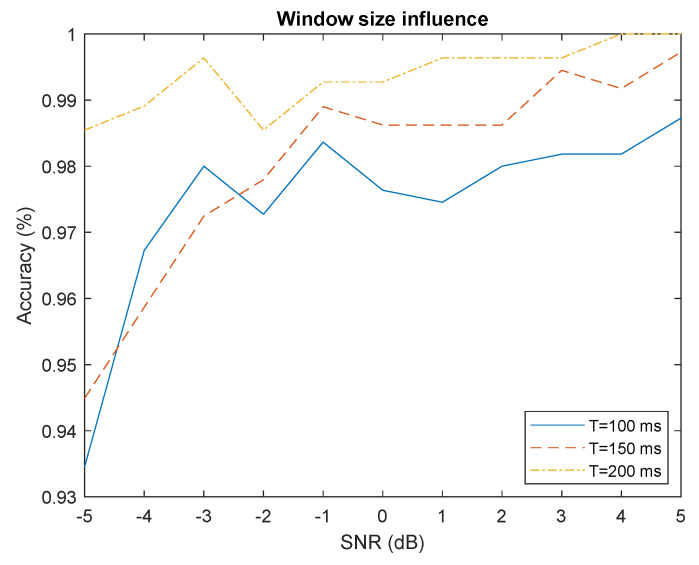
Influence of window size on accuracy.

The window size creates a compromise between performance and robustness to variable RSSI. Increasing the size of the window allows a better estimation of the different statistics (mean, standard deviation) but the packet extraction become more sensitive to variable RSSI, i.e., the signal power varying in time due to movement for example (see Appendix A). We can observe in Figure 8 that increasing window size improves accuracy performance for segment with constant RSSI. Furthermore, increasing the window size also increase the computation complexity because the packet extraction is performed on bigger signal segment.

#### 4.4.2. Processing

**Figure 9:**
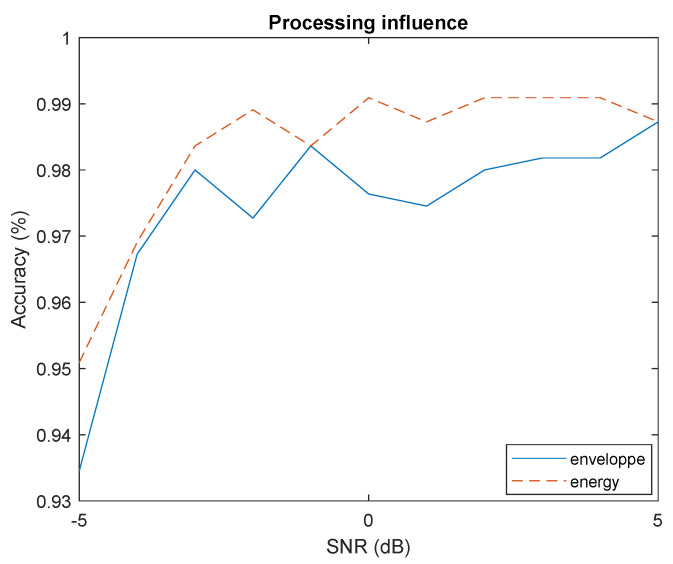
Influence of processing on accuracy.

The processing technique create a compromises between the robustness to co-channel interference and robustness to variable RSSI. Using energy allows to better separate signals but makes the packet extraction method more sensitive to variable RSSI (see Appendix A). The Figure 9 shows the performance of both methods are similar. Furthermore, the energy extraction is simpler to compute compare to absolute value in term of computation complexity.

#### 4.4.3. Threshold

**Figure 10:**
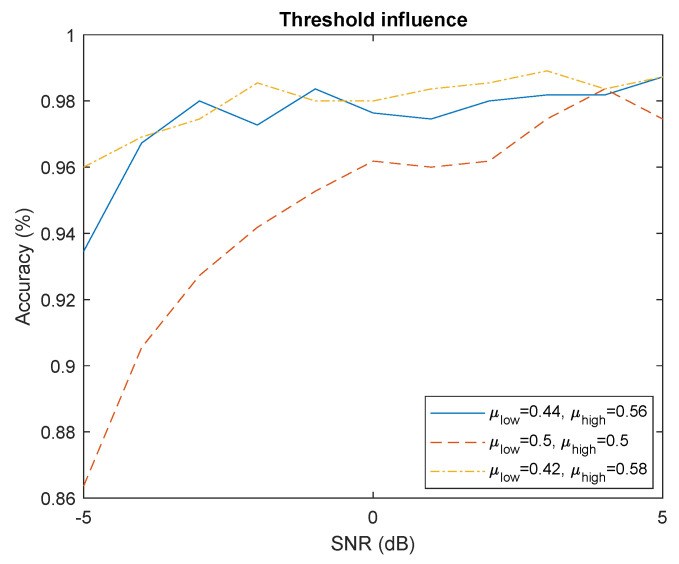
Influence of threshold values on accuracy.

The threshold values create a compromise between noise robustness and robustness to variable RSSI. Increasing ϵ (with $\mu_{low}=0.5-\epsilon$ and $\mu_{high}=0.5+\epsilon$) allows better noise robustness as explained in Section 3.5 but makes the algorithm more sensitive to variable RSSI (see Appendix A). The Figure 10 shows that greater ϵ allows better noise robustness for segment where RSSI is constant. Furthermore, the threshold values does not influence the computation complexity except for ϵ=0. This subcase can be reduce to simple thresholding technique instead of hysteresis technique. Thus, the hysteresis thresholding technique shows better robustness to noise than simpler thresholding techniques based on single threshold.

## 5. Discussion and Perspectives

### 5.1. Discussion

The classification method presented in this paper distinguishes drones by their communication specificities depending of communication requirements and protocol implementation. Particularly, we showed that method based on PLPSF can classify different devices even if they use the same protocol. For example in Section 4.3, we were able to distinguish a Wifi drone link (Bebop) from a smartphone communicating with an AP in WiFi. As already explained, this type of authentication method is a software-based approach being part of non-cryptographic authentication methods [24]. However, the classic software-based approaches work at data link layer such as the methods presented in Section 2.5 using tools such as *tcpdump* or *wireshark*. Thus, these methods are protocol-specific and require to know many information about received signal (modulation, frame format, …). Conversely, PLPSF extracts these communication specificities at the physical-layer without knowing information about received signal.

Thus, PSLPF could be used as a second authentication factor in addition to an other physical-layer authentication method. Particularly, RF Fingerprinting could be interesting as principal physical-layer authentication. These methods consists in authentifying a device using its own hardware impairments such as I/Q imbalance, amplitude clipping and carrier frequency offset among others [26,27,28]. It would allow to combine communication specificities and physical impairments of a specific devices to perform authentication at the physical-layer. An attacker should thus perform features impersonation and protocol impersonation to spoof a specific device, increasing the attack complexity [24]. The main advantage of this combination is that the methods works at different levels of granularity, i.e., the communication level for PLPSF and the impairments level for RF Fingerprinting. Furthermore, PLPSF and RF fingerprinting approaches are complementary in terms of invariance. On one hand, PLPSF exploits communication specificity regardless of device impairments for classification. On the other hand, RF fingerprinting exploits devices impairments regardless of data transmitted for classification.

### 5.2. Perspectives

Even if the algorithms shown noise robustness and invariances to some environmental conditions such as scale invariance and frequency offset, several points can be improved:

- Dataset: Currently, our dataset is limited in terms of classes and recordings. Adding more drones classes and more recordings per drone and thus showing that performances are stable is paramount to prove scalability and generalization of our approach.
- Robust statistics: The features we used for classification algorithm are mean and standard deviation. However, use of robust statistics such as median and interquartile can be interesting because they are less sensitive to outliers.
- Power spectral entropy: We presented a detection approach using PSE, a measure of energy distribution uniformity in the frequency-domain. PSE in time domain could also be used to detect presence of signal to extract packets instead of using hysteresis thresholding and allow better robustness against variable RSSI.
- Other features: The feature used in this approach are principally focusing on temporal aspect. Other types of features can be added to increase accuracy such as frequency or cyclostationary features [8,13].
- Clustering: Packets clustering could be used using packet RSSI, frequency aspects or goniometry. Thus, it could be beneficial to separate control link, video link and telemetry link.
- Real-world implementation: In this study, the central frequencies of communication signals were defined manually. For future implementation, it is necessary to study the use of band scanning techniques compatible with our approach but also to study its hardware implementability.

## 6. Conclusions

We present in this paper a novel signal detection algorithm based on Power Spectral Entropy (PSE) with good detection rate under low SNR. We also present a classification algorithm based on Physical-Layer Protocol Statistical Fingerprint (PLPSF). Besides the fact this classification method exploits the protocol statistical fingerprint at physical-layer instead of data link layer as previously done in other research papers, it also shows interesting invariances to scale (amplitude, phase rotation), frequency offset and good robustness to temporal shift and noise. We also provide statistical analysis and experiments to highlight performances under different environmental conditions. Our method is trained at fixed SNR (5 dB) and evaluate for different SNR values from −5 dB to 5 dB. The records used to train the classifier are different than those used for the test the classifier performances. Furthermore, our dataset included WiFi communications (AP, smartphone, …) in addition to drones communications. This configuration is close to real implementation conditions where SNR is difficult to estimate and others communications signals can be present in analysis band. We also discuss about the interests of using PLPSF as second authentication factor in addition to RF fingerprinting to perform authentication at the physical-layer. Particularly, we present the complementarity between PLPSF and RF fingerprinting methods in terms of granularity level and invariances. Finally, although the methods presented in this article are interesting, several ideas have been proposed to increase performances and robustness such as using packets clustering or perform packets extraction using PSE. Moreover, adding more drone and more recordings could show the scalability and generalization of our approach.

## Figures and Tables

**Table 1:** Drone models.

Drone Model	Protocol
(a) Parrot Bebop	Wifi
(b) Phantom 4 Pro	LightBridge
(c) Mavic 2 Pro	Ocusync 2
(d) Parrot Anafi	Wifi
(e) Syma X5C	Enhanced Shock Burst
(f) Smartphone and AP	Wifi

**Table 2:** Statistical test: Packet length (*p*-value).

Conditions	(1)	(2)	(3)	(4)
(a)	1	0.82	0.99	1
(b)	0.99	**0**	0.66	0.93
(c)	0.99	**0**	0.59	0.99
(d)	0.99	0.87	0.90	0.98
(e)	0.35	**0**	0.22	**0**

**Table 3:** Statistical test: Inter-packet length (*p*-value).

Conditions	(1)	(2)	(3)	(4)
(a)	1	0.99	0.99	1
(b)	0.88	**0**	0.91	0.91
(c)	0.86	**0**	0.35	0.58
(d)	0.99	0.99	0.90	0.99
(e)	0.44	**0**	0.96	0.28

**Figure A1:**
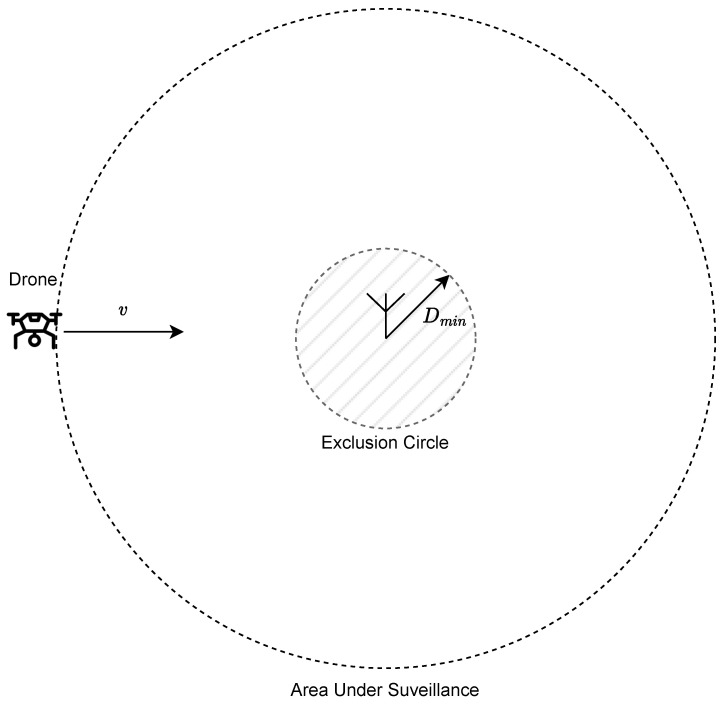
Exclusion circle of monitoring system.

## Data Availability

Not applicable.

## Appendix A. Classification Invariance

The signal consider is the received signal $\tilde{x}\left(t\right)=\tilde{s}\left(t\right)+\tilde{n}\left(t\right)$ where $\tilde{s}\left(t\right)$ is the signal of interest and $\tilde{n}\left(t\right)$ is an additive white gaussian noise. It can be noted that $\forall \;a\in C,a\tilde{n}\left(t\right)$ is an additive white gaussian noise. Moreover, $\forall \;\Delta f\in R,\tilde{n}\left(t\right)e^{j2\pi \Delta ft}$ is an additive white gaussian noise.

### Appendix A.1. Scale Invariance

Considering that signal received is the following:

(A1) $$\tilde{y}\left(t\right)=a\tilde{x}\left(t\right)\;\mathrm{with}\;a=\left|a\right|e^{j\phi_{a}}$$

The phase rotation is removed thanks to absolute value because $|\tilde{y}\left|\left(t\right)=|a|\right|\tilde{x}\left(t\right)|$. Then, the filtering is covariant to multiplication constant because $z_{y}\left(t\right)=|a|z\left(t\right)(=|\tilde{y}\left(t\right)|*h\left(t\right)$).

The obtained low threshold $t_{low}$ is the following:

(A2) $$min_{z_{y}}+\mu_{low}{\bar{z}}_{y}=\left|a\right|\left(min_{z}+\mu_{low}{\bar{z}}_{y}\right)$$

And the high threshold $t_{high}$ is the following:

(A3) $$max_{z_{y}}+\mu_{high}{\bar{z}}_{y}=\left|a\right|\left(max_{z}+\mu_{high}{\bar{z}}_{y}\right)$$

We can observe that the result of comparisons $z_{y}\left(t\right)≶t_{low}$ and $z_{y}\left(t\right)≶t_{high}$ does not depend of the value of |a|. Therefore, the packet extraction process is invariant to scale (homothety and phase rotation).

### Appendix A.2. Frequency Invariance

Considering that signal received is the following:

(A4) $$\tilde{y}\left(t\right)=\tilde{x}\left(t\right)e^{j2\pi \Delta ft}$$

The phase rotation is removed thanks to absolute value because $|\tilde{y}\left(t\right)|=|\tilde{x}\left(t\right)|$, involving that the packet extraction process is invariant to frequency offset.

### Appendix A.3. Variable RSSI

This subsection deals with RSSI variations robustness of our approach. Particularly, we introduce the notion of Exclusion Circle (EC) include in the Area Under Surveillance (AUS) monitored by our system. This EC is a perimeter where the RSSI variations could effect the system performances. Indeed, considering a drone with speed *v* flying toward our system, if the drone enter in the EC, some packets could be not detected due to RSSI variations. The EC also depends on window length $T_{w}$ and high threshold $\mu_{high}$. In this subsection, we will consider a line-of-sight propagation and speed three cases: the high-speed drones case: $v_{max}=80$ m/s, the normal-speed drones case: $v_{norm}=20$ m/s (72 km/h, Phantom 4 Pro) and the low-speed drones case: $v_{min}=5$ m/s (18 km/h: X5C).

#### Appendix A.3.1. Processing: Envelope

For the processing based on absolute value (signal envelope) and based on Friis equation, the EC radius where the method does not perform is equal to:

(A5) $$D_{min}=\frac{T_{w}v_{max}\mu_{high}}{1-\mu_{high}}\Leftrightarrow \frac{\mu_{high}}{D_{min}}=\frac{1}{D_{min}+T_{w}v_{max}}$$

Using $T_{w}=0.1$ and $\mu_{high}=0.56$, the different EC radius are:

- $v_{high}$: 10.2 m
- $v_{norm}$: 2.5 m
- $v_{min}$: 0.6 m

#### Appendix A.3.2. Processing: Energy

For the processing based on energy and based on Friis equation, the EC radius where the method does not perform is equal to:

(A6) $$D_{min}=\frac{T_{w}v_{max}\left(\mu_{high}+\sqrt{\mu_{high}}\right)}{1-\mu_{high}}\Leftrightarrow \frac{\mu_{high}}{D_{min}^{2}}=\frac{1}{{\left(D_{min}+T_{w}v_{max}\right)}^{2}}$$

Using $T_{w}=0.1$ and $\mu_{high}=0.56$, the different EC radius are:

- $v_{high}$: 23.7 m
- $v_{norm}$: 5.9 m
- $v_{min}$: 1.5 m

The EC radius for energy processing are bigger than EC radius for signal envelope. Thus, the signal envelope processing is less sensitive to RSSI variations due to drone movement rather than instantaneous energy processing.

## Appendix B. Detection Invariance

### Appendix B.1. Scale Invariance

Considering that signal received is the following:

(A7) $$\tilde{y}\left(t\right)=a\tilde{x}\left(t\right)\;\mathrm{with}\;a=\left|a\right|e^{j\phi_{a}}$$

The estimate PSD is the following $P_{y}\left(n\right)={\left|a\right|}^{2}P\left(n\right)$. Then the normalisation allow to obtain $p_{y}\left(n\right)=\frac{P_{y}\left(n\right)}{\sum_{n}P_{y}\left(n\right)}\left(=p\left(n\right)\right)$. Therefore, the detection algorithm is invariant to scale (homothety).

### Appendix B.2. Frequency Invariance

Considering that signal received is the following:

(A8) $$\tilde{y}\left(t\right)=\tilde{x}\left(t\right)e^{j2\pi \Delta ft}$$

The estimated PSD $P_{y}\left(n\right)$ will be a shifted version of P(n) by the number of bins corresponding to frequency offset Δf and similarly for $p_{y}\left(n\right)$ and $p_{n}$. Finally, $PSE_{y}=H\left(p_{y}\left(n\right)\right)$ and $PSE=H(p_{n})$ where *H* is the entropy, will be similar because entropy computation is not dependant of central tendency. Therefore, the detection algorithm is invariant to frequency offset.
